# Malaria, climate variability, and interventions: modelling transmission dynamics

**DOI:** 10.1038/s41598-023-33868-8

**Published:** 2023-05-05

**Authors:** Anton Beloconi, Bryan O. Nyawanda, Godfrey Bigogo, Sammy Khagayi, David Obor, Ina Danquah, Simon Kariuki, Stephen Munga, Penelope Vounatsou

**Affiliations:** 1 Swiss Tropical and Public Health Institute, Allschwil, Switzerland; 2 University of Basel, Basel, Switzerland; 3 Kenya Medical Research Institute - Centre for Global Health Research, Kisumu, Kenya; 4 Heidelberg Institute of Global Health (HIGH), Medical Faculty and University Hospital, Heidelberg University, Heidelberg, Germany

**Keywords:** Applied mathematics, Statistics, Malaria, Attribution, Climate-change impacts, Climate-change mitigation

## Abstract

Assessment of the relative impact of climate change on malaria dynamics is a complex problem. Climate is a well-known factor that plays a crucial role in driving malaria outbreaks in epidemic transmission areas. However, its influence in endemic environments with intensive malaria control interventions is not fully understood, mainly due to the scarcity of high-quality, long-term malaria data. The demographic surveillance systems in Africa offer unique platforms for quantifying the relative effects of weather variability on the burden of malaria. Here, using a process-based stochastic transmission model, we show that in the lowlands of malaria endemic western Kenya, variations in climatic factors played a key role in driving malaria incidence during 2008–2019, despite high bed net coverage and use among the population. The model captures some of the main mechanisms of human, parasite, and vector dynamics, and opens the possibility to forecast malaria in endemic regions, taking into account the interaction between future climatic conditions and intervention scenarios.

## Introduction

Despite significant success in reducing the worldwide malaria burden over the last two decades, the disease continues to present a major public health problem. According to the World Health Organization’s (WHO) latest World Malaria Report, there were a total of 241 million malaria cases and 627,000 malaria deaths worldwide in 2020, with most of these cases (95%) and deaths (96%) occurring in the WHO Africa region^1^. Recent models project an overall global net increase in climate suitability and a global net rise in the number of people at risk of malaria^2^; however, other models suggest a shift in the spatial distribution rather than an increase in the global malaria incidence^3,4^. In sub-Saharan Africa, malaria incidence has been decreasing since 2000, which is attributed to the scale-up of malaria interventions. However, in countries with moderate to high malaria transmission, the rate of progress has leveled off since 2015 and, in 2020, the COVID-19 pandemic disrupted malaria services, increasing the malaria burden again in the Africa region^1^.

In this context, the assessment of the impact of climate change on malaria transmission has generated heated discussions^5^, with many studies reporting conflicting results regarding its effect on malaria incidence. The disparities are mostly attributed to differences in modeling approaches, with some studies employing relatively simple biological models that fail to capture the complex nonlinear relationships between environmental determinants and malaria^4,6^, while others apply statistical regression models that mostly ignore important parameters related to transmission of the disease^7,8^. Although most scientists agree that climatic factors play a crucial role in driving the seasonal malaria outbreaks in areas where malaria transmission is low or unstable^9–11^, the relative effects of weather variability in holoendemic transmission settings, where disease incidence is determined not only by external forces, is not fully understood. In endemic settings, malaria incidence is mostly driven by seasonal variations in mosquito population and density, which are largely influenced by local rainfall^9,12^ and temperature patterns^11,13^. Temperature determines the duration of parasite development within the vector, larval development time, vector survival, and mosquito biting rates, as transmission is optimised within specific temperature ranges^14–16^. On the other hand, rainfall contributes to the formation and continuation of mosquito breeding sites, leading to an increase in the vector population^17^. In addition, in high transmission settings, immunity against malaria develops during childhood from repeated infections. This allows individuals to tolerate infections without presenting symptoms^18^.

Several methods have been proposed to explicitly integrate climatic factors into nonlinear process-based malaria models, accounting for immunity and other factors influencing the human-mosquito transmission dynamics^9,11,19,20^. However, most formulations do not account for interactions between climatic and non-climatic factors that are likely to influence the disease incidence, such as malaria interventions, human migration, and other socioeconomic status effects^21^, mainly due to the scarcity of high-quality, long-term data quantifying these factors. The Intergovernmental Panel on Climate Change Fifth Assessment Report (IPCC AR5) shared this ambiguity, providing no clear guidance on the effects of climate change on malaria transmission, while acknowledging a strong link between local weather and malaria incidence^22^.

The Kenya Medical Research Institute (KEMRI) in collaboration with the US Centers for Disease Control and Prevention (CDC) routinely collect data on malaria incidence and mortality, control interventions, vector densities, human migration, and household-related indicators through the health and demographic surveillance system (HDSS) and the population-based infectious disease surveillance (PBIDS) embedded within the HDSS in Siaya County, western Kenya^23,24^. In our earlier work^25^, we used these data to investigate the role of weather variability on the incidence of malaria in the face of intensified malaria control programmes using Bayesian modelling methods. Here, we extend the previous work by applying a nonlinear stochastic transmission model that includes some of the key mechanisms related to malaria dynamics, such as immunity, infectivity, asymptomatic infections, and human migration. In contrast to statistical models, climatic and non-climatic factors are incorporated explicitly into the transmission parameters.

## Results

### Distribution of malaria, control interventions and climatic factors

**Figure 1:**
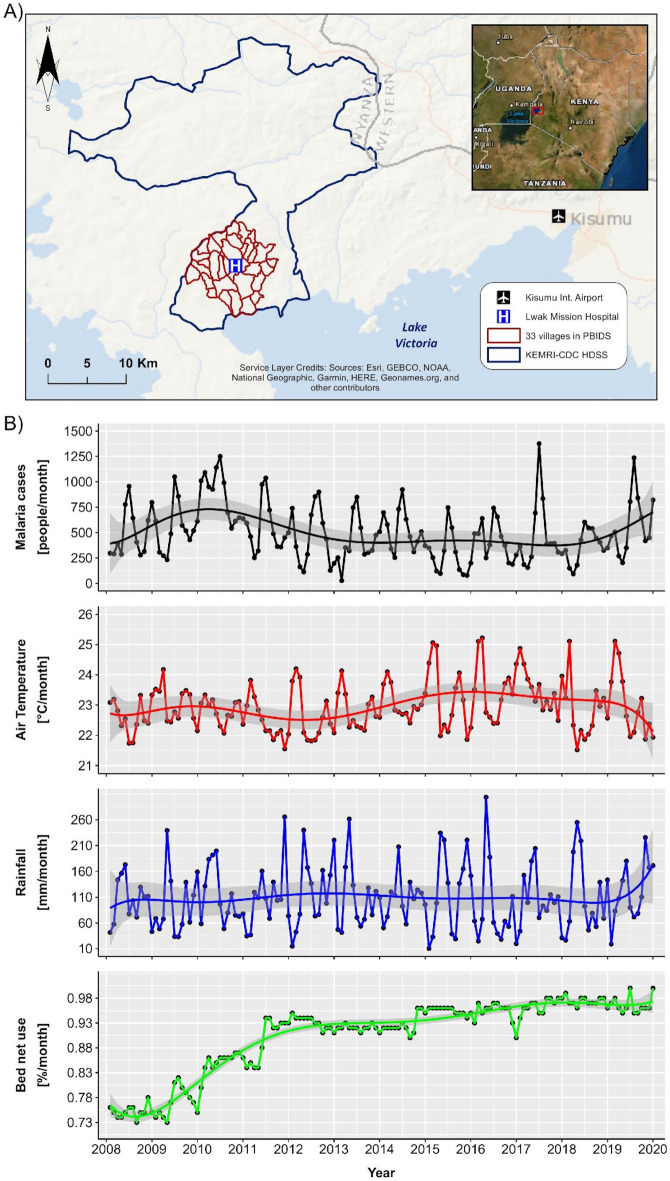
Study area, malaria incidence and covariates. (**A**) The location of Lwak Mission hospital, the 33 villages participating in the population-based infectious disease surveillance (PBIDS), the KEMRI-CDC health and demographic surveillance system (HDSS) area, and the location of the Kisumu international airport. The map was created using ArcGIS Desktop 10.6.1 software (https://www.esri.com). (**B**) Epidemiological evolution of monthly malaria incidence and time-series plots of air temperature, rainfall and bed net use from 2008 to 2019 covering the PBIDS area. B-spline curves with 7 degrees of freedom were fitted to every time-series; shaded areas represent the approximate 95% confidence intervals of each fitted spline.

The PBIDS study area is located next to the shores of Lake Victoria in Siaya County and covers 33 rural villages situated within a 5 km radius from the St. Elizabeth Lwak Mission Hospital (LMH) (Fig. 1A). The region is characterized by a low altitude, and the population is culturally homogeneous with comparable socioeconomic status. During 2008–2019, a total of 71,733 malaria episodes were reported at the LMH. The smoothed plots (spline curves fitted to the time-series) of malaria incidence, air temperature, rainfall, and bed net use are presented in Fig. 1B. Malaria incidence increased by 50% from 2008 to 2010, then declined by 73% until 2015; a resurgence in cases was observed after 2017. Whereas the temporal changes in temperature and precipitation during our study period are less monotonic, there is a clear rise in bed net use coverage between 2008 and 2019 (Fig. 1B). Malaria incidence shows a bi-modal seasonality, with the first and more pronounced peak during May–July, and the second peak during December–January (Fig. 1B). The temporal evolution of air temperature and rainfall reveals a similar bi-modal seasonal pattern but during different months. Rainfall peaked during March through May, with a second peak observed during November, whereas higher temperatures were observed during January–March and September–October. The amount of rain accumulated during the current and previous three months had the highest correlation with malaria incidence; similarly, air temperature had the highest correlation when averaged over the current and prior two months (Fig. S1 in the Supplementary Information (SI)). There was a significant positive correlation between rainfall and malaria incidence (Pearson’s *r*_*xy*_ = 0*.*43, p-value = 7*.*01*e* − 08), whereas the correlation between air temperature and bed net use against malaria was negative, with *r*_*xy*_ =  − 0*.*42 (p-value = 1*.*02*e* − 07) and *r*_*xy*_ =  − 0*.*16 (p-value = 0*.*049), respectively.

**Figure 2:**
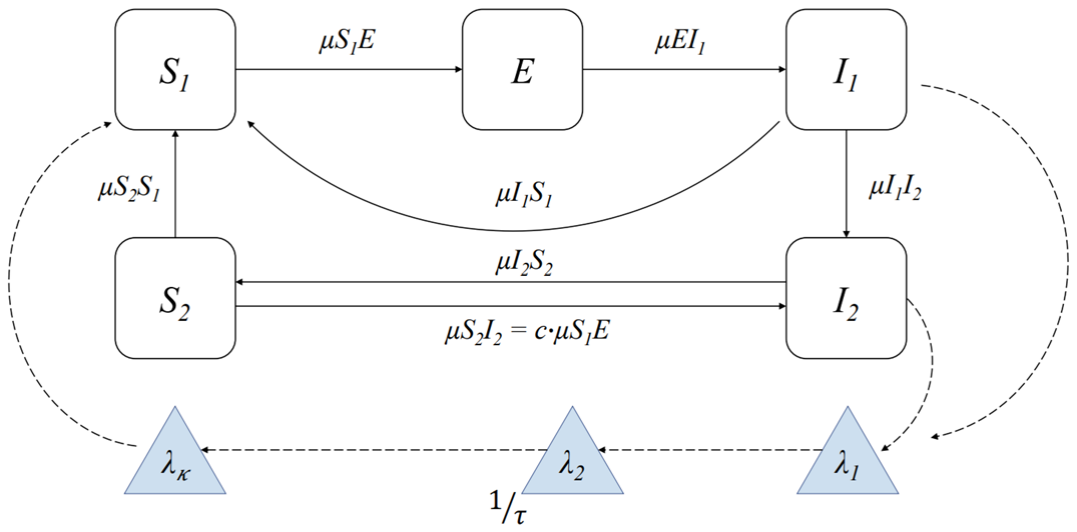
Diagram of the compartmental structure of the model. The model divides the human population into five classes: $S_{1}$-susceptible to infection; $E$-exposed (i.e., carrying malaria parasites which have not yet matured into gametocytes); $I_{1}$-infected symptomatic and gametocytemic (i.e. infectious); $I_{2}$-infected but asymptomatic and with reduced infectivity; and $S_{2}$-recovered and protected from severe disease, but susceptible to mild reinfection. A solid arrow from one class to another denotes the possibility of transition, with rate *µ*. The dotted arrows represent interactions between the human and mosquito stages of the parasite. The chain of the compartments $\left( {\lambda_{1} , \ldots , \lambda_{\kappa } } \right)$ implements a distributed time delay between infections in humans and the force of infection (the per-capita rate of infection) experienced by a susceptible individual, as described in “Methods”. The effect of climate and interventions is included in the transition rate representing transmission $\mu_{{S_{1} E}}$. The model is formalized by the stochastic differential equations (Eq. 1–6).

We carried out likelihood-based inference via iterated filtering^26,27^, a plug-and-play sequential Monte Carlo procedure for calculating maximum likelihood estimates (MLEs). The flow diagram of the model is shown in Fig. 2, and the stochastic differential equations describing malaria transmission are formalized in the “Methods” section. As a first step, we used all the available observations, covering the years 2008–2019, to fit the dynamical model for the transmission of the disease and to estimate the model parameters that maximize the likelihood. The iterated filtering algorithm carries out sequential Monte Carlo while adding stochastic perturbations to the parameters. In subsequent iterations, the intensity of these stochastic perturbations is decreased, and so the likelihood surface is investigated at increasingly local scales.

### Effects of weather variability and bed net use on malaria incidence

We evaluated the effects of the climatic factors and interventions on malaria dynamics by introducing the air temperature (TEMP), rainfall (RAIN) and proportion of bed net use (BEDN) into the model through the force of infection function (Eq. 8 in Methods). The magnitude of the effect for each of these covariates was measured by the corresponding regression coefficients, $b_{T}$, $b_{R}$ and $b_{I}$. These effects were estimated and accounted for seasonality in the data, which was modelled using periodic cubic B-splines as described in “Methods”, and were therefore expected to capture the extra variation in malaria incidence, in addition to the average seasonal variability.

Figure S2 (A) shows the trace plots of the $b_{T}$, $b_{R}$ and $b_{I}$ parameters and the corresponding log-likelihood values of the multimodal function during 250 iterations of the iterated filtering algorithm. Despite the large number of parameters, these three regression coefficients converged in all the models. In particular, starting from random values selected uniformly from the [− 5, 5] interval, all three covariates indicated small variability after the 50th iteration. In fact, the last 100 iterations in Fig. S2B showed that changes in the log-likelihood were minimal and all the regression coefficients fluctuated within the same range of values. As a result, the regression coefficients for rainfall ($b_{R}$) appeared to converge in the region with positive values, whereas the coefficients of temperature ($b_{T}$) and bed net use ($b_{I}$) maximized the likelihood in a negative neighborhood of values. It is worth mentioning that all the three covariates were standardized, and therefore the effects can be directly compared. To evaluate whether the effects of the three covariates were significantly different from zero, we estimated, for each parameter, the profile likelihood curves from which confidence intervals were obtained (as the points at which the profile curve crosses the horizontal line five log-likelihood units below the MLE). The MLE for the rainfall coefficient ($b_{R}$) was positive (Fig. S2C), indicating a significant effect of rainfall in increasing transmission rates. On the other hand, the coefficients of temperature ($b_{T}$) and bed net use ($b_{I}$) indicated a significant negative effect on malaria transmission (Fig. S2D–E).

To evaluate the added value of the seasonal terms, climatic factors, and bed net use to the model fit, we compared the Akaike information criterion (AIC) values^28^, as well as the mean absolute error (MAE), and the root mean square error (RMSE), between the observed and simulated cases for the models with and without these variables. Lower values of each of these metrics represent a better model fit. The results (Table 1) showed that in comparison to the model without covariates (AIC = 1943.6, MAE = 205.8, RMSE = 261.4), the inclusion of splines in the force of infection function highly improved the model fit (AIC = 1902.9, MAE = 144.3, RMSE = 191.1); the inclusion of rainfall, air temperature, and bed net use further decreased the values of all these three metrics (AIC = 1889.0, MAE = 137.3, RMSE = 187.7).

**Table 1:** In-sample predictive performance of stochastic transmission models with different set of covariates included in the force of infection function.

Model	Log-likelihood	Nr. of parameters	AIC	MAE	RMSE
Without covariates	− 954.8	17	1943.6	205.8	261.4
Only with seasonality (i.e. with the *b*_1_–*b*_6_ splines)	− 928.5	23	1902.9	144.3	191.1
With seasonality, AIRT, RAIN and BEDN covariates	− 918.5	26	1889.0	137.3	187.7

*AIC* akaike information criterion, *MAE* mean absolute error, *RMSE* root mean square error, *AIRT* air temperature, *RAIN* rainfall, *BEDN* bed net use.

### Estimated parameters of the malaria transmission dynamics

All the other parameters defining the malaria transmission dynamics of the best fitted model, together with the ranges of the starting values, are shown in Table S1 (in SI). The average time from exposed latent to infected status ($1/\mu_{{EI_{1} }}$) was estimated at 25.2 days, whereas the moving time from the infected class back to susceptible, without developing immunity, was estimated at 20.5 days. As one would expect, the amount of time needed to develop immunity after the infection, as well as to lose this immunity and be susceptible again, takes much longer, with estimates of around 236 and 950 days, respectively. The reporting rate (ρ), i.e., the proportion of the infected population that presented to the hospital with malaria symptoms, was estimated to be ρ = 0.161 (i.e., 16.1%). The relative infectivity of partially immune individuals was rather large (*q* = 0*.*680), whereas the coefficient of reinfection with clinical immunity was low (*c* = 0*.*053). The estimated initial fraction of people in each of the five compartments indicated a larger proportion in those who are recovered and protected from severe disease but are susceptible to mild reinfection ($\left[ {S_{2} } \right]_{0}$ = 54.8%). The lowest proportion of people were initially in the compartment of susceptible individuals ($\left[ {S_{1} } \right]_{0}$ = 3.2%). Although the starting condition for those that were infected and symptomatic ($\left[ {I_{1} } \right]_{0}$ = 28.1%) was larger than that for those asymptomatic and partially immune ($\left[ {I_{2} } \right]_{0}$ = 6.1%), for all the upcoming months (i.e., for $t = 1, \ldots ,{ }144)$ we observed that $\left[ {I_{2} } \right]_{t} > \left[ {I_{1} } \right]_{t}$; i.e., the proportion of those asymptomatic is higher than that of those that present malaria symptoms.

### Model fit and external validation using survey prevalence data

**Figure 3:**
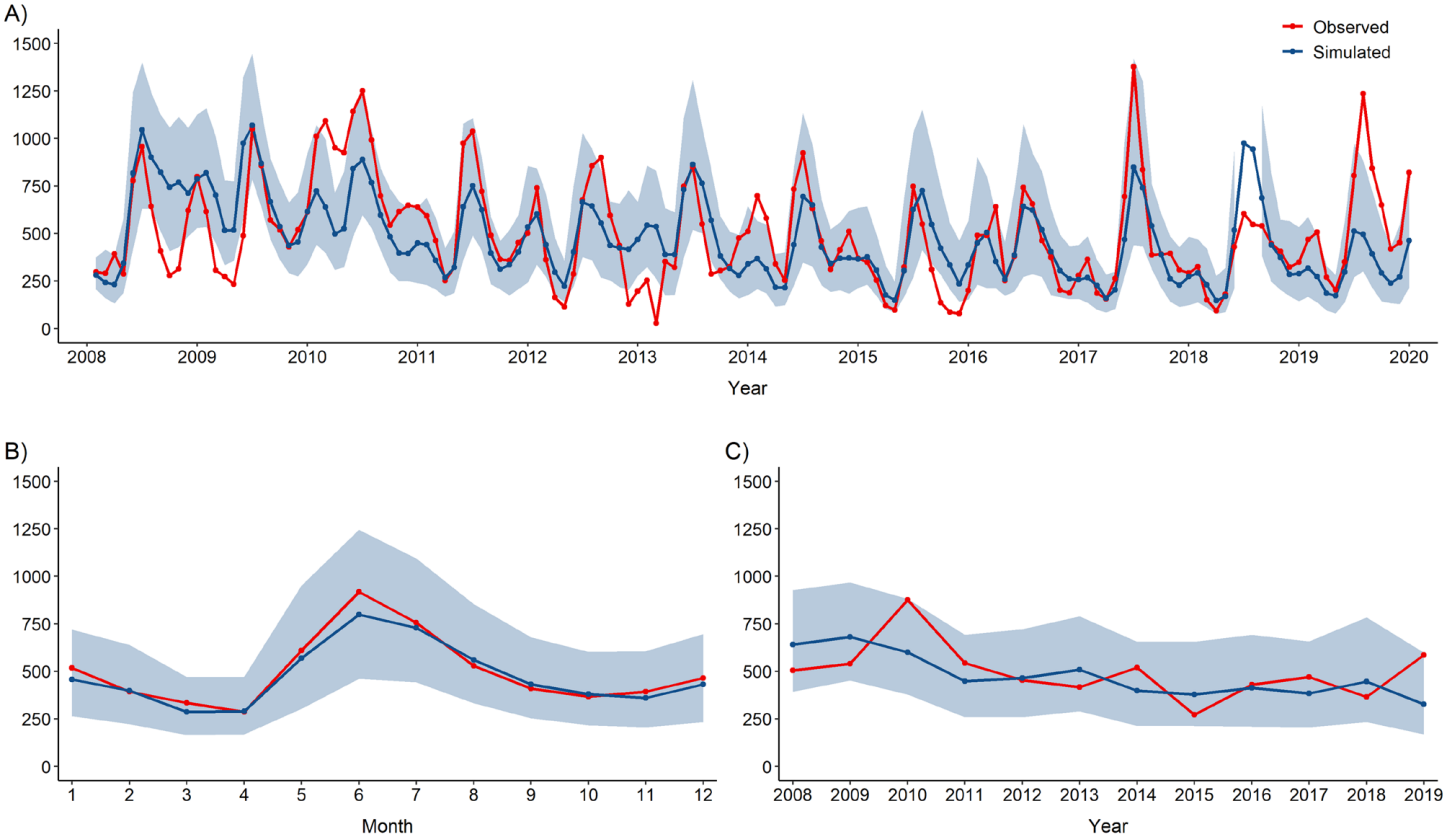
Model fit: comparison of observed and simulated malaria cases. (**A**) Monthly reported malaria cases are shown in red. The medians of 1000 simulations using the best model (i.e., the maximum likelihood estimates for the parameters) are shown in blue, along with their uncertainty (shaded for the 10% and 90% quantiles). (**B**) Intra-annual malaria estimates: comparison of the monthly-averaged malaria observations and simulations during 2008–2019. (**C**) Inter-annual malaria estimates: comparison of malaria observations and simulations averaged for all the months (i.e., January–December) during 2008–2019. Shaded areas denote the uncertainty of the predictions.

Figure 3A depicts the observed (in red) monthly malaria cases and the simulated cases (in blue) using the best-identified model. The simulations were run from the estimated initial conditions, generating all of the time series ahead (i.e., with no readjustment of any parameter at any point in time during the simulation). The median of 1000 simulations indicated good agreement with the observed malaria cases, especially in capturing the average seasonal patterns (Fig. 3B). Figure 3C depicts the inter-annual malaria observations and the corresponding estimates, i.e., the model simulations averaged for all the months (January–December) during 2008–2019.

**Figure 4:**
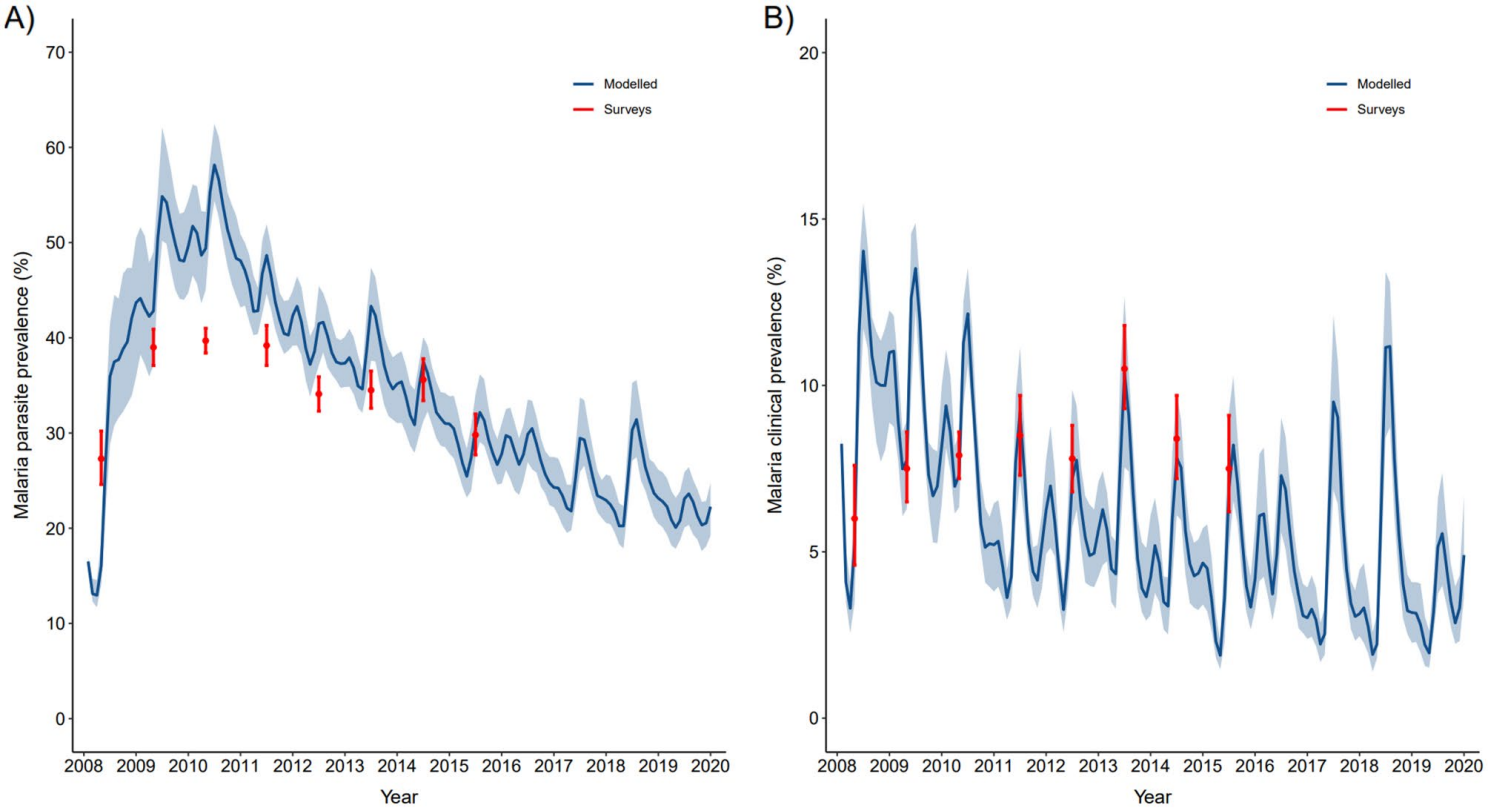
External model validation: comparison of modelled prevalence with data from surveys. (**A**) Malaria parasite prevalence from surveys (mean and 95% confidence intervals), for every available month when they were conducted are shown in red. The median of 1000 simulations of the number of people in *I*_1_ + *I*_2_ compartments for each month of the study period are shown in blue together with their uncertainty (shaded, for the 10% and 90% quantiles). (**B**) Same figure as in (**A**) but for clinical malaria prevalence. Data from surveys (in red) are compared to the simulated number of people in the *I*_1_ compartment of the dynamical model (in blue).

The model’s structure enabled the estimation of clinical malaria (reported fever within 24 h or temperature above 37.5 °C with parasites detected) and parasitic malaria prevalence for each month of the study period. Therefore, we validated our best model by comparing these estimates with the prevalence data obtained from the malaria surveys conducted in the region. To this end, the prevalence survey data covering the period 2008–2015^29^ was compared to the estimates of our process-based transmission model. In particular, for every available month, the malaria parasite prevalence from surveys was compared to the sum of population in $I_{1}$ (infected symptomatic and infectious) and $I_{2}$ (infected asymptomatic with reduced infectivity) compartments for the same months estimated by the model. Similar comparisons were made between survey data on clinical malaria prevalence and the estimated number of people in the $I_{1}$ compartment for the corresponding month. Very good agreement was found between the model simulations and the survey data, especially in the case of the clinical malaria prevalence (Fig. 4).

### Forecasting malaria incidence, taking into account climatic factors and interventions

**Figure 5:**
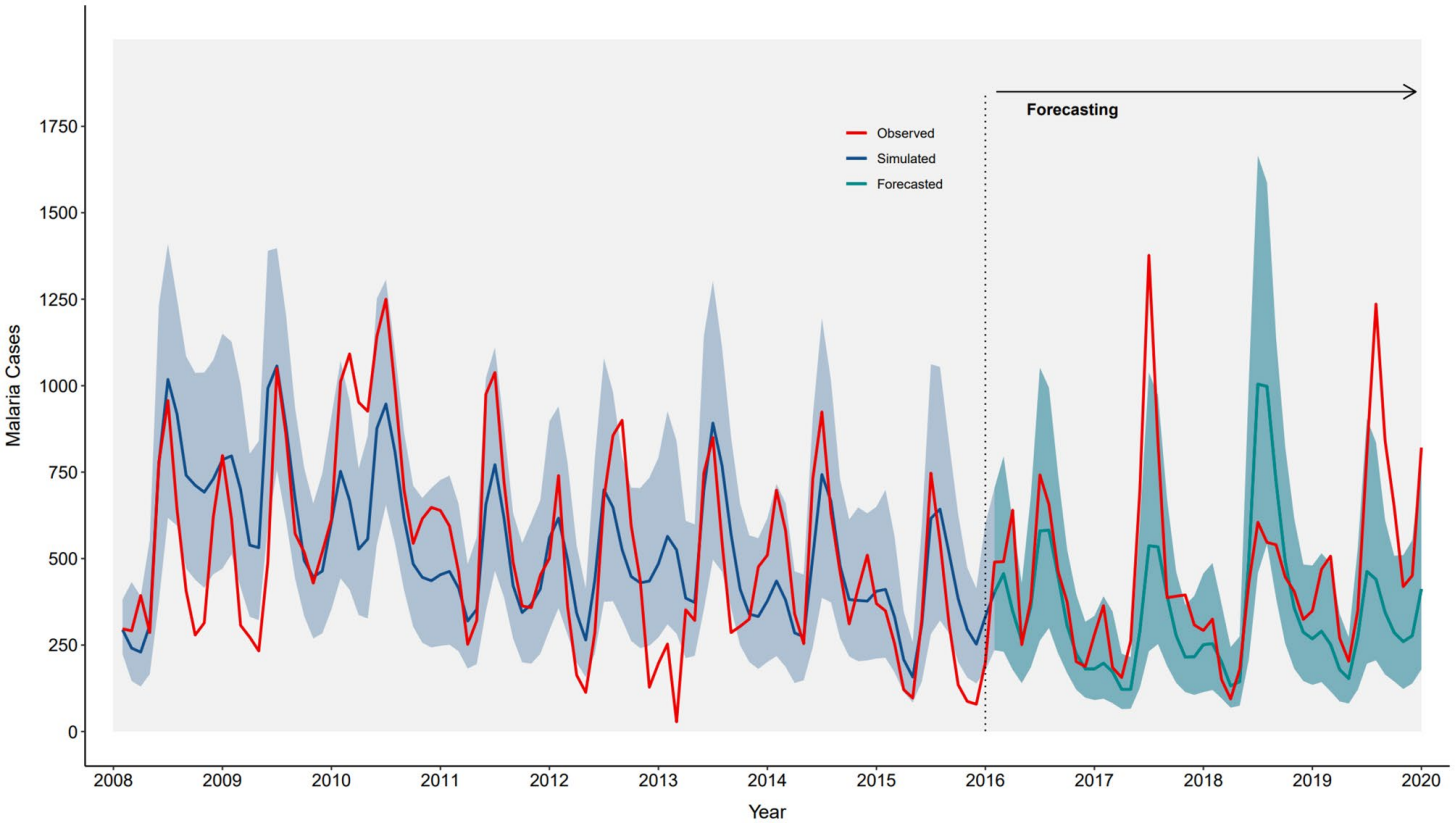
Forecasting malaria cases. The reported cases are shown in red. Median simulated cases (based on 1000 simulations) with the best model are shown in blue for the time period of the training set data (years 2008–2015) together with their uncertainty (shaded, for the 10% and 90% quantiles). Median predictions for the forecasting period (years 2016–2019) are shown in cyan, also with their corresponding uncertainty.

Following the identification of the best model and the validation procedures, we assessed the model’s ability to forecast malaria incidence a few years ahead. To this end, the model was re-fit to the subset of malaria cases in 2008–2015 and the updated estimates were used to forecast monthly incidence for the years 2016–2019, taking into account changes in rainfall, air temperature, and bed net coverage and use during this forecasting period. Figure 5 depicts the agreement between reported (in red) and simulated (in blue) cases during the training period (i.e., years 2008–2015), and compares observed and median forecasted (in cyan) malaria cases 4 years ahead (i.e. for 2016–2019), based on 1000 model simulations, together with the prediction uncertainty (shaded color for the 10% and 90% quantiles). The model appeared to forecast well for 1–2 years ahead, while the predictive ability deteriorated for the last years of the forecasting period.

## Discussion

Model-based malaria surveillance that incorporates weather effects is recognized as an adaptation strategy to address the impacts of climatic variability on malaria outbreaks. Climatic factors are important drivers of malaria transmission, however, other factors such as control interventions and socio-economic development can influence the disease dynamics, too. Here, we developed a stochastic transmission model that allowed quantifying the contribution of climatic and non-climatic factors to malaria incidence in the lowlands of Siaya County in western Kenya during 2008–2019, using the unique population-based infectious disease surveillance dataset. Indeed, despite widespread bed net use and coverage, variations in climatic factors played a key role in driving malaria incidence. The model enabled us to evaluate the relative effects of internal and external factors in malaria epidemiology, to assess the potential degree of predictability emanating from climatic variability, and to generate estimates of some of the main parameters determining malaria dynamics.

We found a significant association between a rise in malaria incidence during a particular month and an increase in the total amount of rainfall accumulated during that month and the 3 months before it. Similar relationships between delayed rainfall and malaria have been estimated in other studies, including investigations in the Rift Valley in Kenya^30^, western Kenya^31^ and Uganda^32^. On the other hand, the model showed that air temperature averaged over the month of reported malaria cases and the 2 months preceding it had a significant negative effect on malaria incidence. Extreme land surface temperature (a proxy of air temperature) was previously linked to a decline in malaria incidence and mortality in western Kenya^33^ and Uganda^32^. According to earlier studies, *Anopheles* mosquitoes (the disease’s vectors) appear to thrive in a temperature range of 22–30 °C ^15,34^. When air temperatures are either low or extremely hot (as they are in this region during the day in the dry seasons), adult mosquitoes are not viable^35^. The findings reported here confirm the results of our previous analysis, in which Bayesian negative binomial models were fitted to a similar dataset^25^, and we found that an increase in daytime land surface temperature (a proxy for air temperature) by 1 °C was associated with a 9% decrease in malaria incidence, whereas an increase in rainfall by 10 mm was associated with a 4% increase in malaria incidence.

In Kenya’s malaria-endemic regions, including our study area, the distribution of bed nets has greatly increased since 2006^36,37^. The fitted transmission model showed that the rise in bed net use had a significant negative association with malaria incidence. Similar findings were made in our previous analysis^25^ and in other studies^38^. Despite the widespread usage of bed nets, a resurgence in malaria cases was seen after 2016. Together with the significant associations estimated for rainfall and air temperature, these findings suggest that this resurgence may be related to weather variability. The comparison between the models with and without the covariates revealed that the inclusion of splines in the force of infection function highly improved the fit of the stochastic transmission model. Adding rainfall, air temperature, and bed net use variables further improved the predictive ability of the model, allowing it to capture extra-variation in the data, besides pure seasonality modelled through splines.

There were 26 parameters to be estimated. Investigating a multimodal likelihood function with such a large number of unknowns may represent a substantial computational challenge, especially for combinations that are weakly identifiable. Allowing some of the transmission parameters to vary in time can result in further over-parametrization, as every additional temporal component means one additional parameter to be estimated. Even with this level of complexity, few parameters related to malaria transmission showed multimodality and poor identifiability, and the model was unable to discriminate between all of these parameters. Nonetheless, some of the findings reported here are consistent with those previously reported or in line with what one would expect in the current malaria setting. For example, according to the WHO, the first *P. falciparum* malaria symptoms usually appear 10–15 days after the infective mosquito bite^1^, whereas the CDC suggests values ranging between 7 and 30 days (https://www.cdc.gov/malaria/about/disease.html), with the shorter periods observed most frequently with *P. falciparum* and the longer ones with *P. malariae.* Our estimate of 25.2 days (1*/*$\mu_{{EI_{1} }}$) is slightly higher, a delay that may be explained by the additional average time to report to the hospital after observing symptoms. The reporting rate was found to be low at 16.1%, and this aligns well with the knowledge about malaria in the region; the fraction of complicated malaria is low, and mostly cases in children will be reported to the hospital. Another reason is the large number of people that developed immunity to malaria exposure throughout their lifespan, as reflected also in the high proportion of those in the $\left[ {S_{2} } \right]_{t}$ compartment estimated during all the months within the study period (i.e., for t = 0,…,144).

It is important to note that this model fits best for the study area during the specified study period and cannot be generalized to other locations or time periods. The operational application of the model in other settings requires re-estimation of all the model parameters. Nonetheless, some transmission dynamics parameters are expected to be similar in analogous malaria settings (i.e., moderate to high transmission environments). When we compare the model fit obtained here to the one of Bayesian negative binomial model that was developed in our earlier work^25^ using a similar dataset, we observe that the latter model has a much better predictive ability. This is to be expected, since requiring a model to be scientifically interpretable may result in a cost in terms of the ability to match data statistically^19^. On the other hand, statistical models disregard the dynamics of malaria transmission and instead assess associations between malaria incidence and climatic and non-climatic factors.

Malaria dynamics are complex and are determined by many other drivers, including indoor residual spraying (IRS), prompt diagnosis, and treatment of malaria using effective artemisinin-based combination therapy (ACT)^39,40^, land-use, urbanization, and socio-economic status, to name a few. However, not all of these factors are applicable to our setting. For example, all patients diagnosed with malaria were treated using ACT, and the IRS was not conducted in the study area during the study period^25^. Our model simulations indicated good agreement with the observed malaria cases, especially in capturing the average seasonal patterns. Nonetheless, some of the parameters unaccounted for in the current analyses may have contributed to the variations in malaria incidence during our study period and could further improve the model fit. Furthermore, the air temperature and rainfall seem to have started changing substantially in the last years of data, and here the model appeared to be fitting the worst; thus, a longer time series may be needed to estimate the contribution of these variables to malaria dynamics in a changing environment.

The structure of the model allowed us to estimate malaria parasite prevalence and clinical (parasites and fever) malaria prevalence for each month during the study period. The model simulations and the survey data showed very strong agreement, particularly for the prevalence of clinical malaria. Finally, we showcased the possibility to forecast malaria in endemic regions (such as the Kisumu area), taking into account the interactions between future climatic conditions and intervention scenarios. The model appears to forecast well for 1–2 years ahead, while the predictive ability deteriorates for the last years of the forecasting period. These modelling approaches could complement early warning systems by informing intervention scenarios that improve responses to weather variability.

Despite the fact that models based on homogeneous populations are often sufficient to describe the major features of disease transmission dynamics^19,41^, patterns of clinical malaria strongly vary by age^42^. In highly endemic areas, the disease burden is greatest in infants and children under the age of 5 years, while in areas of lower transmission, many cases also occur in older children (5 to 14 years) and adults^43^. The developed transmission model was able to reproduce the qualitative dynamics of malaria by capturing some key mechanisms such as the seasonal variation of the disease and was able to disentangle the effects of the local climate and interventions on malaria transmission. However, most facets of malaria heterogeneity, such as the likelihood of being bitten, the development of clinical malaria, immunity development, and mortality rates, vary with age (with children under the age of 5 years bearing the highest burden), and this is a limitation of the current model. For example, in our previous work^25^, we showed that both climatic factors and control interventions have different effects on malaria incidence in different age groups. It might be tempting to add as much biological detail as possible when creating dynamic transmission models of biological systems. As a result, certain parameters or parameter combinations may be weakly identifiable by the available data or may not converge. However, while the fitted malaria model may appear simplistic in that it ignores some critical malaria attributes, such as age heterogeneity and superinfection, adding these components to the model can make it computationally infeasible due to the multimodality of the likelihood function and the weak identifiability of certain parameters. Here, we concentrated on findings that are not sensitive to identifiability problems, at least for the estimation of the parameters of interest (i.e., the effects of climatic and non-climatic factors on malaria incidence). Nonetheless, we would like to acknowledge the fact that these limitations need to be taken into consideration when modelling malaria transmission dynamics, if deemed possible, and this will be part of our future work.

## Methods

### Study area

The Kenya Medical Research Institute (KEMRI) in partnership with the US Centers for Disease Control and Prevention (CDC) have conducted a population-based infectious disease surveillance (PBIDS) since 2005, in Asembo-Siaya County^37^. The PBIDS is embedded within the health and demographic surveillance system (HDSS) and covers approximately 30,000 people residing in 33 rural villages within a 5 km radius from the St. Elizabeth Lwak Mission Hospital (LMH) in close proximity to Lake Victoria (Fig. 1). The population characteristics have been previously described^24,25^.

### Malaria incidence data

In Siaya, malaria is endemic with year-round transmission. Here, we analysed data collected from LMH between January 2008 and December 2019. All patients visiting LMH with symptoms of febrile illness had their finger prick blood taken for microscopy to determine whether they had malaria. Following the guidelines of the Ministry of Health, all individuals who tested positive were treated with artemisinin-based combination therapy (ACT). We calculated monthly malaria incidence by dividing the monthly number of malaria cases by the total monthly person follow up time in years. We did not include children under six months of age in this analysis; it is deemed that malaria is uncommon in this age group, as the newborns are protected by maternal antibodies throughout this time^44^.

### Bed net use data

We evaluated the existing malaria interventions through the PBIDS by estimating the proportion of individuals who reported using a bed net the night before the interview. For temporal alignment with the malaria incidence data, we aggregated the bi-weekly household visits data^23^, collected between January 2008 and April 2015, by month. The number of visits to each home was reduced to two per year afterwards, but the data collection instruments remained the same. The procedure to access these data is described in the “Data availability” statement.

### Climatic data

We extracted monthly near-surface (at 2 m) air temperature (in °C) at ~ 11 km^2^ spatial resolution from the ERA5-Land-ECMWF Climate Reanalysis dataset^45^. Daily rainfall was accessed from the Climate Hazards Group InfraRED Precipitation with Station data^46^ (CHIRPS) at ~ 5.5 km^2^ resolution; we have calculated the monthly amount of precipitation (in mm/month) by summing up the daily data over each month. For spatial linkage with the monthly malaria incidence, we averaged both products within the HDSS area using the Google Earth Engine (GEE) API^47^. We used the remotely sensed products since there was no weather station within the study area during our study period; the closest station was situated approximately 60 km away, at the Kisumu international airport (Fig. 1A).

### Dynamic malaria transmission model

We fitted a stochastic malaria transmission model based on prior modifications of the compartmental susceptible-exposed-infected-recovered (SEIR) model, with two susceptible classes distinguishing those who acquired immunity from past infection and are asymptomatic upon reinfection, as well as two infected (symptomatic and asymptomatic) classes^19,20^. In particular, the human population was divided into five categories: $S_{1}$-susceptible to infection; $E$-exposed (i.e., carrying malaria parasites that have not yet matured into gametocytes); $I_{1}$-infected symptomatic and gametocytemic (i.e., infectious); $I_{2}$-infected but asymptomatic and with reduced infectivity; and $S_{2}$-recovered and protected from severe disease but susceptible to mild reinfection. The diagram illustrating the compartmental structure of the model is shown in Fig. 2. With $\mu_{XY}$ we denote the rate of transmission from class *X* to class *Y*, for *X, Y* ∈ {$S_{1}$, $E$*,*$I_{1}$*,* $I_{2}$*,* $S_{2}$}; e.g. the rate $\mu_{{EI_{1} }}$ corresponds to the transition from exposed ($E$) to infected and symptomatic ($I_{1}$), and therefore $1/\mu_{{EI_{1} }}$ represents the average time of development of the parasite within the human host. Our model incorporates the possibility of failing to build any protective immunity following infection by transitioning directly from $I_{1}$ to $S_{1}$ without passing via $I_{2}$ and $S_{2}$. This may be the case for children that have not yet built immunity or adults who have lost immunity. The transition from $S_{2}$ to $I_{2}$ might be regarded as reinfection with clinical immunity, i.e., reduced symptoms that do not lead the patient to seek medical care^48^. We assume that $\mu_{{S_{2} I_{2} }}$ = *c* ·$\mu_{{S_{1} E}}$, such that 0 ≤ *c* ≤ 1, implying that the susceptibility to infection is reduced by a factor *c* that accounts for the fact that these people had a history of malaria, and thus the susceptibility to infection is expected to be smaller.

We do not explicitly model mosquito abundance, survival, or parasite development; instead, we use a delayed equation for the force of infection $\mu_{{S_{1} E}}$, which accounts for the parasite’s extrinsic incubation period ($\tau$) within the mosquito, during which time it completes its sporogonic cycle, as has been done in other studies^19,20^. Thus, a mosquito stage $\lambda$ represents the latent force of infection, capturing the likelihood of successful transmission from human to human together with a distributed delay. We use the high-quality western Kenya HDSS dataset, which includes information on the rates of births $\left( {B\left( t \right)} \right)$, deaths $\left( {D\left( t \right)} \right)$, as well as the in $\left( {M_{in} \left( t \right)} \right)$, and out $\left( {M_{out} \left( t \right)} \right)$ migration. We consider all the newborns to go into the susceptible (*S*_1_) compartment after six months from birth. For migration, we assume that influx (and outflux) is equally proportional in (and out of) the five compartments. We also assume that people die with the same probability in each compartment and therefore subtract the reported monthly deaths equally proportional to the number of people in each compartment. We denote the total population with $P\left( t \right)$ and ensure that $P\left( t \right) = S_{1} \left( t \right) + E\left( t \right) + I_{1} \left( t \right) + I_{2} \left( t \right) + S_{2} \left( t \right)$.

The corresponding system of stochastic differential equations is given by:

1 $$\frac{{dS_{1} }}{dt} = B\left( t \right) + M_{in} \left( t \right)\frac{{S_{1} }}{P\left( t \right)} - \mu_{{S_{1} E}} S_{1} + \mu_{{I_{1} S_{1} }} I_{1} + \mu_{{S_{2} S_{1} }} S_{2} - \left[ {M_{out} \left( t \right) + D\left( t \right)} \right]\frac{{S_{1} }}{P\left( t \right)}$$

2 $$\frac{dE}{{dt}} = M_{in} \left( t \right)\frac{E}{P\left( t \right)} + \mu_{{S_{1} E}} S_{1} - \mu_{{EI_{1} }} E - \left[ {M_{out} \left( t \right) + D\left( t \right)} \right]\frac{E}{P\left( t \right)}$$

3 $$\frac{{dI_{1} }}{dt} = M_{in} \left( t \right)\frac{{I_{1} }}{P\left( t \right)} + \mu_{{EI_{1} }} E - \mu_{{I_{1} S_{1} }} I_{1} - \mu_{{I_{1} I_{2} }} I_{1} - \left[ {M_{out} \left( t \right) + D\left( t \right)} \right]\frac{{I_{1} }}{P\left( t \right)}$$

4 $$\frac{{dI_{2} }}{dt} = M_{in} \left( t \right)\frac{{I_{2} }}{P\left( t \right)} + \mu_{{I_{1} I_{2} }} I_{1} + \mu_{{S_{2} I_{2} }} S_{2} - \mu_{{I_{2} S_{2} }} I_{2} - \left[ {M_{out} \left( t \right) + D\left( t \right)} \right]\frac{{I_{2} }}{P\left( t \right)}$$

5 $$\frac{{dS_{2} }}{dt} = M_{in} \left( t \right)\frac{{S_{2} }}{P\left( t \right)} + \mu_{{I_{2} S_{2} }} I_{2} - \mu_{{S_{2} S_{1} }} S_{2} - \mu_{{S_{2} I_{2} }} S_{2} - \left[ {M_{out} \left( t \right) + D\left( t \right)} \right]\frac{{S_{2} }}{P\left( t \right)}$$

The force of infection or transmission rate at the current time *t* is defined as:

6 $$\mu_{{S_{1} E}} \left( t \right) = \mathop \smallint \limits_{ - \infty }^{t} \gamma \left( {t - s} \right)\lambda \left( s \right)d\Gamma \left( s \right)$$

where $\gamma \left( {t - s} \right)$ is a delay Gamma distribution (for the duration of the parasite life cycle inside the mosquito plus vector survival), with mean delay $\tau$ and variance $\tau^{2} /\kappa$, i.e. a $Gamma(\kappa , \kappa /\tau$):

7 $$\gamma \left( t \right) = \frac{{\left( {\kappa /\tau } \right)^{\kappa } t^{\kappa - 1} }}{\Gamma \left( \kappa \right)}exp\left( { - \kappa t/\tau } \right)$$

and $\lambda \left( s \right)$ is the force of infection at a previous time $s$ when the mosquito bites an infectious human:

8 $$\lambda \left( t \right) = \frac{{I_{1} \left( t \right) + qI_{2} \left( t \right)}}{P\left( t \right)}\tilde{\beta } exp\left\{ { b_{T} TEMP + b_{R} RAIN + b_{I} BEDN + \mathop \sum \limits_{i = 1}^{{n_{s} }} b_{i} s_{i} \left( t \right)} \right\}\frac{d\Gamma }{{dt}}$$

where q ∈ [0, 1] represents the fraction of transmissibility from asymptomatic infections in partially immune individuals $I_{2}$, relative to those from full-blown infections (i.e. $I_{1}$); $\tilde{\beta }$ is a dimensional constant set as $\tilde{\beta } = year^{ - 1}$ to give $\mu_{{S_{1} E}} \left( t \right)$ units of $t^{ - 1}$; $b_{T}$, $b_{R}$ and $b_{I}$ are the regression coefficients for the temperature (TEMP), rainfall (RAIN) and bed net use (BEDN), respectively; the coefficients {$b_{i}$} model the seasonality of the disease and correspond here to a periodic cubic B-spline basis {$s_{i} \left( t \right), i = 1, \ldots , n_{s} \}$, constructed using $n_{s}$ evenly spaced knots. The amount of rain accumulated during the current and previous three months had the highest correlation with malaria incidence and therefore was used in the models. Similarly, the air temperature had the highest correlation when averaged over the current and prior two months (Figure S1 in the Supplementary Information (SI)). All the predictors were standardized to allow direct comparison of the covariate effects.

The gamma-distributed transition $Gamma\left( {\kappa ,\kappa /\tau } \right)$ between the latent $\lambda \left( t \right)$ and the current $\mu_{{S_{1} E}} \left( t \right)$ force of infection was chosen to allow a differential representation that facilitates numerical solution^49^. The integral in Eq. (6) can be replaced by the $\kappa$-dimensional Markovian system, $\lambda_{1} \left( t \right), \ldots , \lambda_{\kappa - 1} \left( t \right),\lambda_{\kappa } \left( t \right)$ $( \equiv \mu_{{S_{1} E}} \left( t \right)$):

9 $$d\lambda_{1} \left( t \right)/dt = \left( {\lambda - \lambda_{1} } \right)\kappa \tau^{ - 1}$$

10 $$d\lambda_{i} \left( t \right)/dt = \left( {\lambda_{i - 1} - \lambda_{i} } \right)\kappa \tau^{ - 1} \;{\text{for}}\;i = 2, \ldots , \kappa$$

After experimenting with different choices of $\kappa$, we fixed $\kappa = 2$. The final term in Eq. 8 models the environmental noise and represents unaccounted variation beyond the covariates and seasonality; $\Gamma \left( t \right)$ denotes a Gamma process with stationary independent increments such that $\Gamma \left( t \right) - \Gamma \left( s \right)\sim Gamma\left( {\left[ {t - s} \right]/\sigma^{2} ,\sigma^{2} } \right)$. The rationale behind choosing a Gamma noise is that of keeping the term $\lambda \left( t \right)$ positive at all times; since the Gamma process is increasing, its derivative ($d\Gamma \left( t \right)/dt)$ is non-negative at all times (and all of the states have to be non-negative). We solve this system numerically via the Euler method^50^ with a time-step of 1 day.

Let {$t_{n} , n = 1, \ldots , N\}$ denote the times of the $N$ observations. We suppose that the model is initialized at some time $t_{0} < t_{1} ,$ and define the number of all new latent cases in the $n$-th interval to be $C_{n} = \mathop \smallint \limits_{{t_{n - 1} }}^{{t_{n} }} \mu_{{EI_{1} }} E\left( s \right)ds$. Thus, the variable $C_{n}$ represents the accumulated new infections (or incidence) sampled in our simulations of the transitions from $E$ to *I*_1_ during a given interval of time (here, a month). To consider that the number of confirmed cases (*y*_n_) is under-reported and measured with error, we introduce a measurement model given by a negative binomial distribution so that $y_{n} \left| {C_{n} ~\sim NegBin\left( {\rho C_{n} ,\psi ^{2} } \right)} \right.$ with overdispersion ψ and reporting rate ρ. Here, ρ is a constant ranging from 0 to 1, representing the proportion of infected, symptomatic patients who present to the hospital.

The model is fitted to the time series of malaria cases between 2008 and 2019, using a sequential Monte Carlo method based on particle filtering for likelihood maximization by iterated filtering^26,27^ and implemented in the R-package pomp^51^. This approach enables partial observations of the system as well as consideration of both process and measurement noise. In-sample predictions were generated by simulating from the best model, i.e., using the Maximum Likelihood Estimates (MLE) parameters. These estimates (including their uncertainty intervals) are provided by the filtering algorithm and are used to initialize the simulations for the predictions. Since the model is stochastic, we generated 1000 simulations and obtained the median and 10–90% percentiles for the monthly cases from 2008 to 2019.

We validated the best model by comparing estimated prevalence for each month with the prevalence data obtained from surveys conducted within the HDSS. The all-age surveys of malaria prevalence covering the period 2008–2015 were carried out by randomly selecting compounds within the HDSS as described in Khagayi et al.^29^. Parasite prevalence was defined as the proportion of participants in each village that had malaria by blood smear microscopy out of all the participants from the same village who were tested for malaria. Clinical malaria prevalence was defined as the proportion of participants in each village who had malaria parasites of any density by microscopy in combination with either a reported fever in the previous 24 h or a temperature of 37.5 °C and above out of all those tested. Both measures of transmission (parasite and clinical malaria prevalence) were compared to the estimates obtained from our model. In particular, the malaria parasite prevalence from the surveys, for every month when they were conducted, was compared with the sum of the population in *I*_1_ and *I*_2_ compartments for the same months as estimated by the model. Similarly, the clinical malaria prevalence data from the surveys was compared with the number of people in the *I*_1_ compartment during the corresponding month.

We further evaluated the ability of the developed model to forecast monthly malaria incidence a few years ahead. To this end, the model was re-fitted to the subset of malaria cases in 2008–2015 and the updated estimates were used to forecast monthly incidence for the years 2016–2019, taking into account changes in rainfall, air temperature, and bed net coverage and use during this period.

### Ethics statement

The HDSS and PBIDS study protocols were reviewed and approved by the KEMRI scientific and ethics review unit (SSC # 1801 and 2761) and CDC’s institutional review board (CDC IRB # 3308 and 6775). All patients, or their parents or legal guardians if they were minors, provided written informed consent. Compound heads also provided written informed consent for the household-based evaluation.

## Supplementary Information

Supplementary Information.

## Data Availability

The data used in this study are available from the Kenya Medical Research Institutes’ Institutional Data Access / Ethics Committee for researchers who meet the criteria for access to confidential data. The PBIDS and HDSS data can be accessed by contacting gbigogo@kemri.go.ke, dobor@kemri.go.ke or munga_os@yahoo.com.
