# Supplementary material for: Malaria, climate variability, and interventions: modelling transmission dynamics
Source: Sci Rep. 2023 May 5;13:7367. doi: 10.1038/s41598-023-33868-8 (PMC10161998; doi:10.1038/s41598-023-33868-8)
Supplement: Supplementary file 1 — Supplementary Information. [file 41598_2023_33868_MOESM1_ESM.docx]

# Supplementary Information

# Supplement to:

# Beloconi A., Nyawanda B.O., Bigogo G., Khagayi S., Obor D., Danquah I., Kariuki S., Munga S.,

# & Vounatsou P. Malaria, climate variability, and interventions: modelling transmission dynamics.

# Contents:

# Figure S1: Malaria cases, rainfall and air temperature

# Figure S2: Effect of climatic and bed net use variables on malaria transmission

# Table S1: Fitted parameters of the best malaria stochastic transmission model with splines, rainfall, air temperature and bed net use covariates.


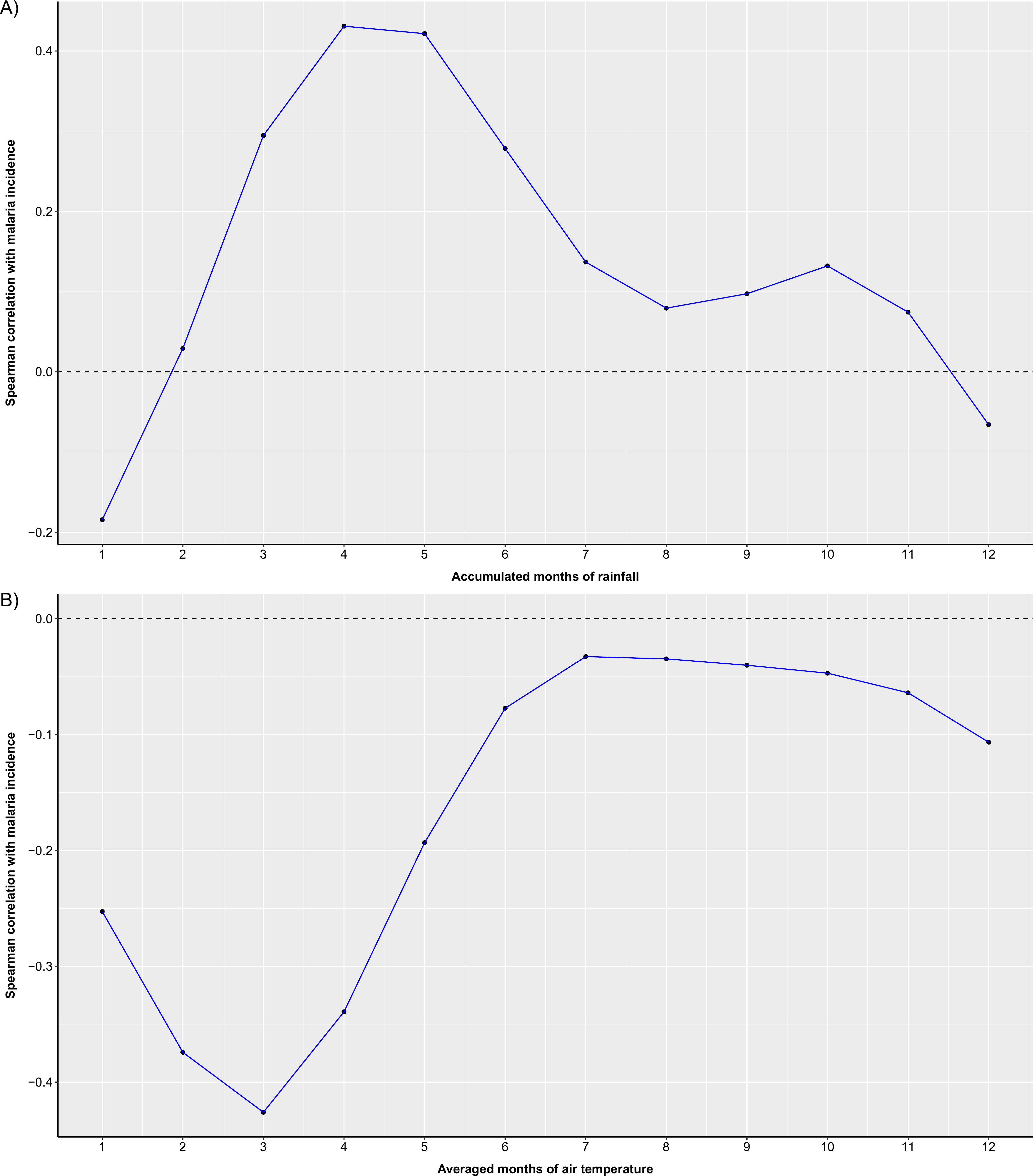


Figure S1: **Malaria cases, rainfall and air temperature. A.** Correlation between accumulated rainfalls in different time windows preceding the month of reported malaria cases. A maximum positive correlation is observed when rainfall is accumulated over 4 months (i.e. during the current and previous three months). **B.** Correlation between averaged air temperatures during different time windows preceding the month of reported malaria cases. A maximum negative correlation is observed when air temperature is averaged over 3 months (i.e. during the current and previous two months).

#
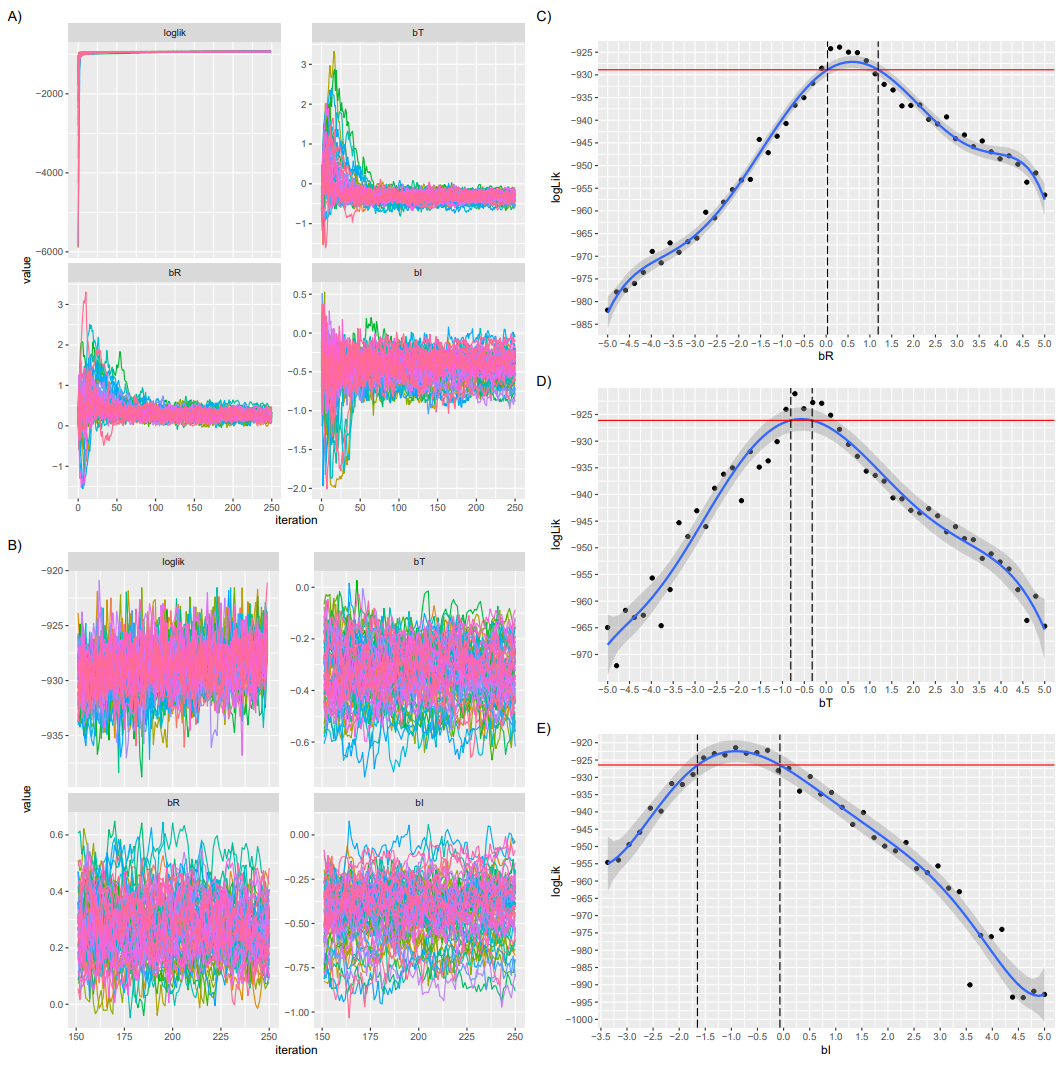


Figure S2: **Effect of climatic and bed net use variables on malaria transmission. A.** Convergence of the regression coefficients $b_{T}$, $b_{R}$ and $b_{I}$ corresponding to the temperature, rainfall and proportion of bed net use variables, respectively. The plot shows the changes in these parameters and the log-likelihood (loglik) during the 250 iterations of the iterative filtering algorithm. Starting from random values within a [-5, 5] interval, all the three regression coefficients indicate small variability after 50th iteration. **B.** Same plot as in **A** but zoomed in the last 100 iterations. **C, D, E.** Profile likelihood curves of regression coefficients for air temperature (**C**), rainfall (**D**), and proportion of bed net use (**E**), and the corresponding confidence intervals (computed as the points at which the profile curve crosses the horizontal line five log-likelihood units below the maximum likelihood estimate).

Table S1: Fitted parameters of the best malaria stochastic transmission model with splines, rainfall, air temperature and bed net use covariates.

| **Parameter** | **Description** | **Starting Values** | **Estimate** |
| --- | --- | --- | --- |
| Temperature ($b_{T}$ ) | Air temperature (2m above ground) regression coefficient | [-5, 5] | -0.278 |
| Rainfall ($b_{R}$) | Accumulated Rainfall (CHIRPS) regression coefficient | [-5, 5] | 0.284 |
| Intervention ($b_{I}$) | % Bed net use (PBIDS/HDSS) regression coefficient | [-5, 5] | -0.517 |
| Spline coefficient *b*_1_ | B-spline regression coefficient | [-10, 10] | 3.430 |
| Spline coefficient *b*_2_ | B-spline regression coefficient | [-10, 10] | 0.382 |
| Spline coefficient *b*_3_ | B-spline regression coefficient | [-10, 10] | 6.076 |
| Spline coefficient *b*_4_ | B-spline regression coefficient | [-10, 10] | 1.479 |
| Spline coefficient *b*_5_ | B-spline regression coefficient | [-10, 10] | 2.182 |
| Spline coefficient *b*_6_ | B-spline regression coefficient | [-10, 10] | 2.624 |
| 1*/µ_EI_*1 | Time from exposed to infected | [1, 365] | 25.2 days |
| 1*/µI*1*S*1 | Time from infected to susceptible | [1, 365] | 20.5 days |
| 1*/µI*1*I*2 | Time from symptomatic to asymptomatic | [1, 365] | 236.3 days |
| 1*/µI*2*S*2 | Time from asymptomatic to recovered | [1, 365] | 645.8 days |
| 1*/µS*2*S*1 | Time from recovered to susceptible | [1, 365] | 950.3 days |
| *ψ* | Dispersion parameter of the observation noise | [0, 0.5] | 0.179 |
| *σ* | Standard deviation of the process noise | [0, 0.5] | 0.244 |
| *ρ* | Reporting rate | [0, 1] | 0.161 |
| *q* | Relative infectivity of partially immune individuals | [0, 1] | 0.680 |
| *c* | Coefficient of reinfection with clinical immunity | [0, 1] | 0.053 |
| [*S*_1_]_0_ | Initial susceptible population | [0%, 100%] | 3.2% |
| [*E*]_0_ | Initial exposed population | [0%, 100%] | 7.7% |
| [*I*_1_]_0_ | Initial infected symptomatic population | [0%, 100%] | 28.1% |
| [*I*_2_]_0_ | Initial infected asymptomatic population | [0%, 100%] | 6.1% |
| [*S*_2_]_0_ | Initial protected population | [0%, 100%] | 54.8% |
| [*λ*_1_]_0_ | Initial values for the latent force of infection | [0, 10] | 2.55 |
| [*λ*_2_]_0_ | Initial values for the latent force of infection | [0, 10] | 0.25 |
